# Finger flexion to extension ratio in healthy climbers: a proposal for evaluation and rebalance

**DOI:** 10.3389/fspor.2023.1243354

**Published:** 2023-11-23

**Authors:** Marine Devise, Léo Pasek, Benjamin Goislard De Monsabert, Laurent Vigouroux

**Affiliations:** ISM, CNRS, Aix-Marseille University, Marseille, France

**Keywords:** muscle force ratio, muscular imbalance, finger, sport climbing, training

## Abstract

**Introduction:**

Finger strength is a key factor in climbing performance and is highly dependent on the capacity of the finger flexor muscles. The majority of finger-specific training therefore focuses on improving such capabilities by performing finger flexion contraction during hanging exercises on small holds. However, greater strength in the finger flexors causes an imbalance with the extensor muscle capacities. Such an unfavourable imbalance may be detrimental to finger strength and could possibly lead to an increase in the risk of finger injury. The aim of this study was to develop an easily implementable method to assess the flexor-to-extensor imbalance and evaluate the effects of different training on it.

**Methods:**

Seventy-eight experienced climbers were tested to assess their maximum finger flexion strength (MFS), maximum finger extension strength (MES) and MFS/MES ratio. Fifty-two of them were randomly assigned to one of three training regimens: intermittent static flexion at 80% MFS (TFlex; *n* = 11), intermittent static extension at 80% MES (TExt; *n* = 10), intermittent repetition of alternating flexion and extension (TPaired; *n* = 11) or no specific training (CTRL; *n* = 20). They trained twice a week for four weeks on a hangboard. Before and after training, force data were recorded on a force-sensing hangboard and MFS, MES and the MFS/MES ratio were compared using ANCOVA.

**Results:**

The mean value of the MFS/MES ratio was 6.27 (confidence interval: 5.94–6.61) and the extreme ratio was defined above 8.75. Concerning the training intervention, no difference was observed in the CTRL group between pre- and post-tests. MFS improved significantly in the TFlex (+8.4 ± 4.4%) and TPaired (+11.9 ± 10.5%) groups, whereas MES increased significantly in the TExt group (+41.4 ± 31.3%). The MFS/MES ratio remained statistically stable among all groups (+0.9 ± 17.5% in TFlex, −1.9 ± 16.1% in TPaired), although the TExt group showed a decreasing trend (*p* = 0.1; −27.8 ± 17.6%).

**Discussion:**

These results showed that only the extensor-based training had an effect on finger extension strength and the potential to rebalance the MFS/MES ratio.

## 1. Introduction

Rock climbing has become immensely popular over the past 20 years with nearly 45 million climbers worldwide in 2019 according to the International Federation of Sport Climbing (IFSC). During climbing, practitioners apply force on their feet and pull with their arms to move upwards (1–3). In these movements, the athletes exert high-force intensities with the fingers on holds of different shapes and sizes (4). Climbers thus need very high finger strength to be able to hold onto the thinnest possible holds. Previous studies have shown that the maximum finger strength was 18%–27% greater in climbers compared with non-climbers (5–7). Finger strength is also highly related to the climbing grade level (8, 9), i.e., expert climbers have greater strength than skilled climbers, who in turn have greater strength than novices.

The effort exerted on the fingertips induces high mechanical loadings on the musculoskeletal system of the upper limbs, including wrist, forearm, elbow, shoulder and shoulder girdle regions. When grasping a hold, the muscular forces generated produce net joint moments in the hand joints that allow the specific hand/finger position to be maintained and produce the external force applied to the hold. Under the influence of these loadings, the climbers' hands develop many adaptations which may be bony (10), ligamentous and/or muscular (11). Since finger flexors are the main agonist muscle for climbing grips (12, 13), it is logical that the climbers develop flexor muscle capacities over time and throughout years of practice. Vigouroux et al. (14) used a biomechanical model and an overall hand testing procedure to determine that the finger flexor force capabilities are 37% higher in climbers compared with non-climbers. When focusing on the antagonist muscle groups, the estimation of muscle forces during climbing grip showed that finger extensor muscles are also highly engaged (15). Moreover, EMG parameters indicated that extensors fatigued at the same intensity as flexors (7). In spite of this, the extensors' force capacities of climbers estimated in the study of Vigouroux et al. (13) did not show the same strengthening as flexors and were comparable with those of non-climbers, and even tended to be lower. These findings showcased a higher flexor-to-extensor finger force ratio (the ratio of the agonist to antagonist muscle force capacities) in climbers (6.1 on average) compared with non-climbers (3.7 on average), with a difference of 67% between the two populations. These observations raise concerns regarding the optimum balance between flexor and extensor (agonist and antagonist) strengths necessary for both maximizing finger performance and practising climbing safely.

The agonist-to-antagonist balance of strength has been widely investigated to quantify the co-contraction in different joints such as knee, ankle, shoulder or wrist in various populations (16, 17). It is thought that the role of an imbalance of the joint musculature, i.e., values that deviating from previous references, may be a possible cause of pathologies by reducing the stability of the joint. Thus, the imbalanced flexor-to-extensor ratio in climbers' finger muscles raises doubts about their ability to balance the entire chain of segments from the forearm to the fingertips by maintaining stability and effectively controlling the joints to enhance finger strength. Peters (18) and Phillips et al. (19) suggest that the potential risk factor for finger injury could be attributed to the imbalance resulting from underdeveloped finger extensor muscles. However, since no measurements or values were obtained in these studies, this link remains unsubstantiated in the current state-of-the-art. Nonetheless, exploring this potential source of injury is crucial, given the prevalence of upper extremity injuries, particularly those to the fingers, during climbing (20). For example, joint instability and overuse injuries, especially in the wrist, are the potential injuries that could be caused in part by an unfavourable flexor-to-extensor ratio, as is the case with the shoulder (21).

Since finger grip strength is related to climbing performance (8), climbers and trainers tend to focus on finger-specific training to constantly improve their finger flexion strength, mostly by hanging on a fingerboard or campus board (22). However, this training strategy (i.e., using the finger- and campus board) does not necessarily reduce the imbalance of the flexor-to-extensor ratio. Some authors (18, 19) have proposed to regulate this imbalance by including finger extension exercises in a training routine. This idea is of interest since, for full hand grip, Shimose et al. (23) have shown that the training of wrist extension significantly improved both the wrist extension strength by about 91% and the hand grip strength by about 3% in an untrained healthy population. Similarly, elbow extension training was found to increase both the elbow extension (+8.5%) and flexion (+5.8%) strength in untrained women (24). Therefore, antagonist-based training seems to be potentially beneficial both for strength enhancement and for reducing the finger flexor-to-extensor imbalance with a greater increase in extension/antagonist strength than in flexion/agonist strength.

To summarize, even if no proof of links between flexor-to-extensor balance, injuries and finger strength has been found, many climbers and coaches already train extensor muscles in the perspective of improving finger strength or preventing injuries. Nevertheless, such practice faces several unknowns. The first is that the only available method (14) to evaluate the flexor-to-extensor ratio is too complex to be used daily and the climbers thus have no means to appreciate the level of imbalance. The second is that no training methods to improve this imbalance have been quantified and evaluated. The climbers and trainers are therefore unaware of the effectiveness of extensor training. The overall objective of this study was thus to investigate the issue of antagonist muscle adaptation in climbers from the point of view of muscular capabilities, and was twofold. The first was to propose an easily implementable test to assess the flexor-to-extensor imbalance of climbers’ fingers and to establish a reference database. To this aim, the finger flexion and the finger extension strengths were measured to compute the ratio in a sample of climbers. The results obtained were used to estimate the normal distribution of values among climbers and classify them to help diagnose climbers. Correlation with the climbing grade level was tested to examine a link between imbalance and grade level. We hypothesized that (i) the extensor capacities would not correlate with the climbing grade level, unlike the trend for flexor capacities and thus that (ii) the flexor-to-extensor imbalance would increase with the climbing grade level. The second objective was to provide an effective training protocol to modify this ratio by quantifying the effect of different types of extensor training. We hypothesized that flexor-based training would increase flexor strength, whereas extensor-based training would enhance both flexor and extensor strength, allowing a rebalance of the flexor-to-extensor ratio.

## 2. Methods

### 2.1. Participants

Seventy–eight climbers were assessed (22 women and 56 men, 25.7 ± 6.7 years old, 64.9 ± 8.6 kg, 173.0 ± 9.0 cm) for the finger strength profile (including flexor strength, extensor strength and flexor-to-extensor ratio). Participants' climbing levels ranged from intermediate to elite on the International Rock Climbing Research Association (IRCRA) scale (25), with an average of 20.3 ± 4.3 in their self-reported best red-point grade in the past six months. They had all practised climbing (indoors and/or outdoors) at least twice a week for the past two years, and had had no upper limb injuries in the previous six months. In addition, although carrying out regular practice, no climber had followed a specific training protocol lasting several weeks in the six months prior to this study. All participants volunteered and signed an informed consent form. The study was conducted with the formal approval of the CERSTAPS Ethics Committee.

### 2.2. Procedures

The 78 climbers were tested in a pre- and post-format described below. Of the initial sample, 52 climbers (15 women and 37 men, 25.7 ± 6.9 years old, 65.4 ± 8.5 kg, 172.9 ± 9.7 cm; 19.0 ± 4.3 in their best red-point grade) participated in the experiment by following a specific training protocol. The climbers were randomly assigned into four different training protocols. Based on previous research done on finger-specific training in climbing (26, 27), the training program lasted 4 weeks (weeks 1–4) with 2 sessions per week and started the week after the pre-test session (week 0). A post-test session, identical to the pre-test, was performed the week after the end of the training sessions (week 5) (22). All climbers were instructed to continue their climbing activities normally and regularly outside of the study throughout week 0 to week 5.

### 2.3. Pre- and post-test sessions

The pre- and post-tests consisted in measuring the finger flexion and extension strengths using a hangboard (SmartBoard, Peypin d'Aigues, France) instrumented with force sensors (strain gauges) measuring the vertical force applied on the holds (0.8 N accuracy, 50 Hz acquisition, 0–4,000 N range of measurement). The associated app provided real-time feedback on the force exerted, allowing precise modulation of the force intensity during training. Before each test session, participants first underwent a 20-min standardized warm-up and familiarization with the instrumented hangboard, consisting of muscular awakening (scapular retractions, shoulder and wrist circles, finger grips, etc.) traverses and specific exercises (pull down, push up with fingers) on the hangboard with increasing intensity. Then, they performed the tests, which consisted of assessing maximum finger flexor strength (MFS) and maximum finger extensor strength (MES). Four trials were performed in each condition (two warm-up trials and two maximum trials). Participants were asked not to train or climb the day before the experiment and to be ready to perform as much as possible. The same experimenter was present during all test sessions (before and after training), checked the correct execution of the tasks for each test and verbally motivated the participants to ensure maximum performance.

#### 2.3.1. Flexor strength test

**Figure 1:**
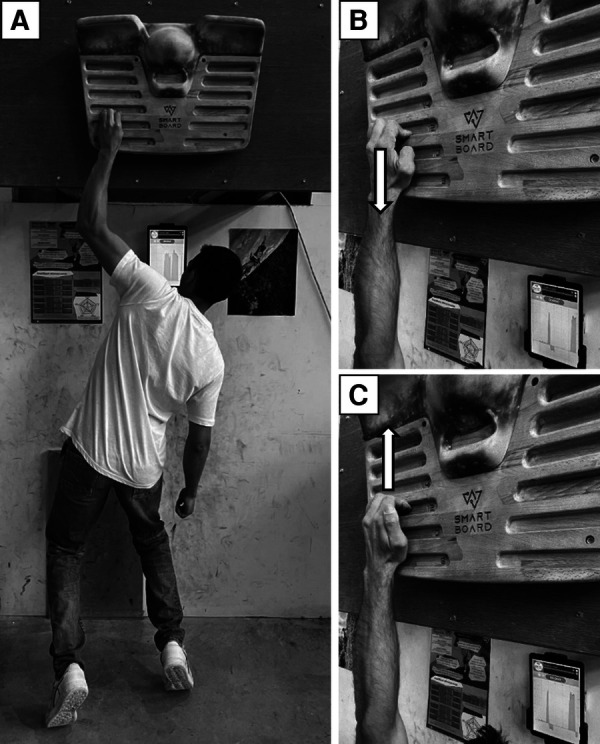
Illustrations of the position of the climbers on the SmartBoard (**A**), with a zoomed-in view of the fingers for flexion (**B**) and extension (**C**) during the test and the training sessions (with both hands during training). Arrows indicate the direction of the applied force.

Participants were asked to exert a maximum force downwards with the palmar aspect of the fingers of both hands on a 12 mm hold for 6 s with the right hand and then with the left hand. When pulling, the participants kept their feet on the ground and tried to hang with a maximum amount of weight (Figures 1A,B). One participant was able to hang with his entire body weight with one hand. To allow him to exert a greater force, we loaded him with a 20 kg mass attached to his harness so that he could not hang completely. Each participant self-selected the grip type (either half-crimp or slope grip), although thumb use was not allowed, and each climber was required to use the same grip throughout all test sessions. Self-selection of grip type was done to ensure maximum finger flexion performance for each participant, allowing a condition to be tested in which the finger flexors were activated as much as possible. For each trial, the MFS was evaluated as the mean of the total force exerted by both hands and recorded by the instrumented hangboard during the 4-s window centred on the force peak. The absolute value was displayed directly on the app in newtons (*N*) and was considered as the MFS. MFS was also normalized by body weight. Two trials, separated by a 3-min rest period, were evaluated and the best was selected for the analysis.

#### 2.3.2. Extensor strength test

On an inverted 12 mm hold with light padding to avoid pain, participants had to exert a maximum force upwards using the dorsal aspect of the distal phalanges of their fingers, with the intention of extending them, while being prevented from doing so by the top of the hold (Figure 1C). The fingernails were positioned almost horizontally and parallel to the hold surface. The distal interphalangeal joints did not touch the hold at any point. Participants were asked to adopt a finger position close to a half-crimp grip, whereby the distal interphalangeal joints were slightly flexed, while the proximal interphalangeal joints were highly flexed (>40°), thereby preventing the intrinsic muscles from extending at the distal finger joints. The thumb was not in contact with the hold. Both hands were tested successively for 6 s. As with MFS, MES was evaluated from the same absolute mean value of the total force exerted by both hands that was displayed by the app in *N*. MES was also normalized by body weight. Two trials with a 3-min rest in between were evaluated and the best was selected for the analysis.

Following the recording of MFS and MES, the flexion-to-extension ratio (MFS/MES ratio) was computed by dividing the MFS by the MES. A ratio superior to 1 means that MFS is higher than MES.

### 2.4. Training sessions

The participants were first randomly divided and followed three different types of training (TFlex, TExt, TPaired described below) and a control group (CTRL). Taking into account dropouts, other participants were recruited so that the climbing level, gender and age matched between the groups. TFlex focused on training finger flexion strength only, TExt focused on finger extension strength only, while TPaired aimed to train both flexion and extension strengths simultaneously. The same grip types (in flexion and extension) were used for all training sessions as for the test sessions. The CTRL group (*n* = 20) did not follow any specific training and only continued their normal climbing activity. The three training sessions were best matched in terms of the duration of effort, the perception of the load during the pre-test. In this sense, 10 s of effort in flexion appeared as an equivalent perception of effort of 5 s for extensors. We therefore added a set of repetitions for TExt compared with TFlex in order to achieve, at best, a similar duration. For TPaired, the duration of effort was longer than for the others, as we took into account the time needed to switch from flexion to extension.

#### 2.4.1. Flexor training protocol (TFlex)

Participants (*n* = 11) in the TFlex group followed a flexor training protocol consisting of reproducing the “F80” training presented by Devise et al. (22). To sum up, this training consisted of exerting finger flexion isometric contractions at an intensity of 80% MFS with both hands on the 12mm-hold of the hangboard. They completed a series of 12 repetitions with a 10-s effort phase followed by a 6-s rest phase. If the participants were unable to achieve 70% MFS during the hanging phase, the series was stopped. The force level was controlled throughout the protocol by the visual feedback and carefully adjusted by off-loading with the feet on the ground or conversely using an additional load attached to a harness. Three sets were performed, with 8 min of recovery time between each set.

#### 2.4.2. Extensor training protocol (TExt)

The participants (*n* = 10) in the TExt group followed an extensor training protocol equivalent (number of sets, repetitions and intensity) to the TFlex training: it consisted of exerting finger extension isometric contractions with both hands, alternating a 5-s push phase (in the same position as for the extensor strength test) and a 6-s rest phase, for a maximum of 10 repetitions or, if the participants were unable to apply 70% MES, the series was stopped. Four sets were performed, separated by a 2-min recovery period.

#### 2.4.3. Paired flexor and extensor training (TPaired)

A final group (*n* = 11, TPaired group) followed a flexor-extensor training protocol based on agonist-antagonist paired (APS) training, a method involving the alternation of agonist and antagonist exercises (28). Thus, the current training consisted of exerting finger flexion at 80% MFS intensity with both hands, followed immediately by finger extension at maximum intensity. During the extension phase, the finger position was identical to that of the extensor strength test. Participants completed a series of 12 repetitions of an 8-s flexion phase, followed by a 5-s extension phase on the inverted 12 mm-hold, then followed by a 6-s rest phase. When any climber was unable to achieve 70% MFS during the hanging phase, the series was stopped. Three sets were performed, with an 8-min recovery period between each set.

### 2.5. Statistics

Data are reported as mean ± SD. Descriptive statistics were used to verify whether the basic assumption of normality was correct for all the variables studied. As we were testing a mixed gender group, we first tested for the presence of any differences between men and women using ANCOVA (with climbing level as a co-variate) or non-parametric ANCOVA when variables did not follow a normal distribution. Then, to categorize the participants, the results of the MFS/MES ratio were divided into eight classes allowing them to be listed from “very low” to “extreme” ratio. The number of classes was determined using Sturges' rule, appropriate for *n* < 200 (29). Considering a normal distribution, the value of *Z*-score for a probability of <0.05 was computed and the confidence interval of the MFS/MES ratio was computed. Pearson's correlations were used to observe the relationship between the climbing level and the different parameters (MFS, MES, and the MFS/MES ratio). The effects of training on MFS, MES and the MFS/MES ratio were assessed by comparing the training groups (CTRL, TFlex and TExt and TPaired) over time (pre- and post-tests) using a 2-factor repeated-measures ANCOVA (Time × Group, with climbing level as a co-variate), with Tukey *post-hoc* analysis and power (1-*β*) when ANCOVAs were significant. In addition, effect sizes (partial eta squared, *η*²) were computed and were defined as small for *η*²>0.01, medium for *η*²>0.09 and large for *η*²>0.14 (30).

## 3. Results

### 3.1. Finger strength profile

The finger strength profile variables for all participants are presented in Table 1. Analysis of the data performed after dividing the groups based on gender indicated that MFS and MES were higher in men than in women when expressed in N but when normalized to body weight, no differences were observed between men and women in MFS (*p *= 0.82) and MES [*F*(1,75) = 0.004; *p *= 0.95; *η*²=0.00]. The MFS/MES ratio (*p *= 0.16) was also similar between men and women.

**Table 1:** Results (mean ± SD) of maximum finger flexor (MFS) and extensor (MES) strengths in absolute values and normalized to body weight (BW) and flexor-to-extensor ratio (MFS/MES ratio) for all participants, in men and women during the pre-tests and correlation of variables with climbing grade level.

		Absolute strength (*N*)	BW normalized strength	*r*
MFS	Total	791 ± 178	1.25 ± 0.24	0.68*
	Men	854 ± 159	1.28 ± 0.23	0.67*
	Women	632 ± 117^a^	1.16 ± 0.25	0.65*
MES	Total	130 ± 30	0.21 ± 0.04	0.04
	Men	136 ± 29	0.20 ± 0.04	0.03
	Women	113 ± 24^a^	0.21 ± 0.05	0.23
MFS/MES Ratio	Total	6.27 ± 1.5		0.52*
	Men	6.44 ± 1.43		0.43*
	Women	5.86 ± 1.63		0.65*

^a^ Significant difference with men (*p *< 0.001).

* Significant correlation with climbing grade level (*p *< 0.001).

A significant correlation between the climbing grade and MFS and the MFS/MES ratio was observed but there was no correlation between the climbing grade and MES. The same results were observed in men and women for MFS, MES and the MFS/MES ratio.

**Figure 2:**
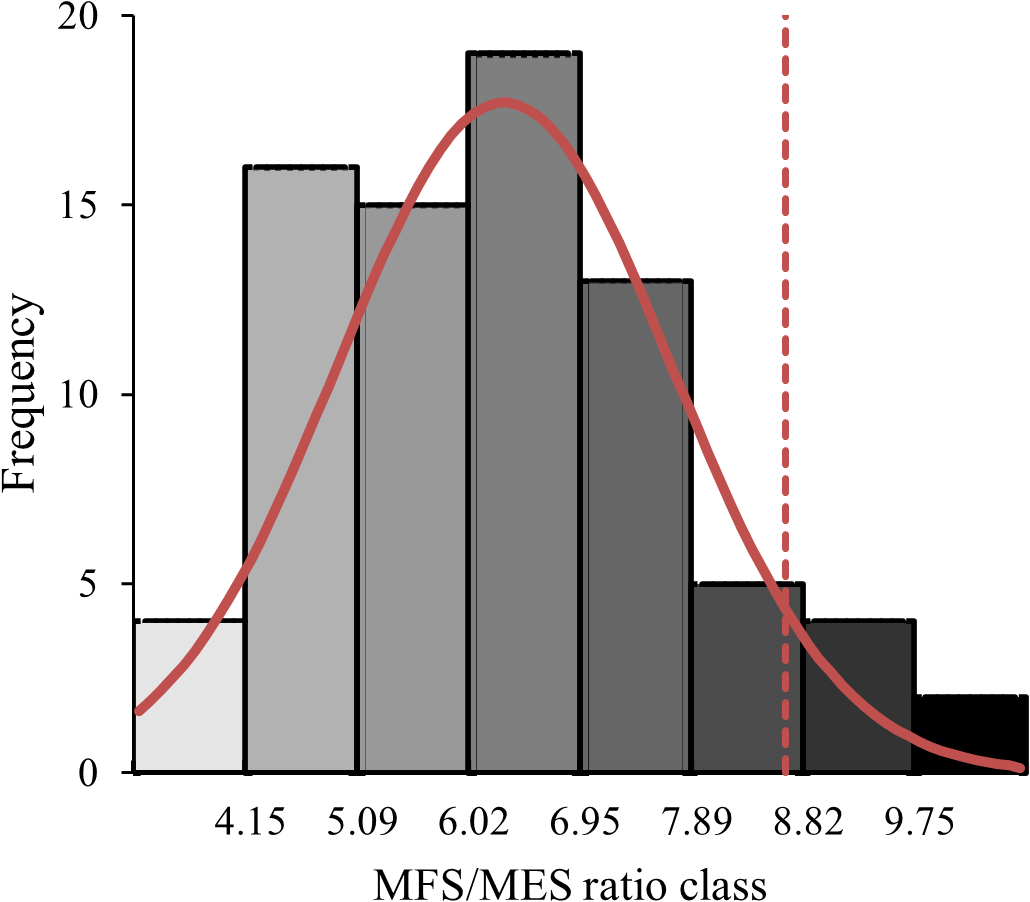
Histogram of frequency distribution of the number of participants (men and women combined) by flexor-to-extensor ratio (MFS/MES ratio) classes. The red curve represents the normal distribution with the vertical red dotted line representing the upper limit for *p* < 0.05.

The eight MFS/MES intervals are shown in Figure 2. Since no significant difference was observed between men and women, the histogram was based on pooled data and made it possible to classify intervals from “very low ratio” to “extreme ratio”. The ratios of the lowest class were less than 4.15 while the extreme ratios were above 9.75, meaning that the finger flexors were 9.75 times stronger than the finger extensors in this class. The mean value was 6.27 and the confidence interval was within the range of 5.94 and 6.61. The *Z*-score for a *p *< 0.05 probability corresponded to a value of 8.75.

### 3.2. Training effects

The anthropometric data and climbing ability of participants involved in the different training groups are summarized in Table 2. No statistical differences were observed between groups for all variables.

**Table 2:** Descriptive characteristics (mean ± SD) of the participants of each group (control, CTRL; flexor training, TFlex; extensor training, TExt; flexor-extensor training, TPaired).

	CTRL	TFlex	TExt	TPaired	*p*-value
Age (y)	28.5 ± 9.1	24.3 ± 5.3	27.2 ± 7.5	23.3 ± 4.7	0.15
Height (cm)	172.4 ± 9.5	176.8 ± 9.0	168.3 ± 9.6	174.7 ± 9.6	0.31
Body mass (kg)	64.0 ± 8.8	67.5 ± 6.4	62.7 ± 8.3	67.0 ± 10.3	0.61
Red-point grade	18.6 ± 4.8	21.2 ± 3.2	16.6 ± 4.7	19.3 ± 3.5	0.09

Red-point grade means climbing a sport route after inspecting and practising it, and represents the most difficult grade achieved in the past 6 months, converted to the IRCRA scale.

*p*-values represent results of the one-way ANOVA comparing the four groups.

MFS, MES and the MFS/MES ratio results before and after training according to the different groups are presented in Table 3. There were significant Time × Group interaction effects for MFS [*F*(3,47) = 7.4; *p *< 0.001; *η*²=0.32; 1-*β*=0.91], MES [*F*(3,47) = 6.0; *p *= 0.001; *η*²=0.28; 1-*β*=0.86] and a tendency in the MFS/MES ratio [*F*(3,47) = 1.37; *p *= 0.099; *η*²=0.12; 1-*β*=0.48]. Post-hoc tests revealed that MFS was greater after training than before in the TFlex and TPaired groups, and MES was greater after training than before in the TExt group, and the MFS/MES ratio seemed to decrease after training in the TExt group.

**Table 3:** Mean ± SD results of maximum finger flexor (MFS) and extensor (MES) strength normalized to body weight (BW) and flexor-to-extensor strength ratio (MFS/MES ratio) before (pre) and after (post) training, according to the groups (control, CTRL; flexor training, TFlex; extensor training, TExt; flexor-extensor training, TPaired).

		CTRL	TFlex	TExt	TPaired
MFS/BW	Pre	1.17 ± 0.26	1.25 ± 0.15	0.99 ± 0.19	1.21 ± 0.14
	Post	1.21 ± 0.27	1.36 ± 0.17*	0.95 ± 0.18	1.34 ± 0.13*
	Difference (%)	3.6 ± 9.8	8.4 ± 4.4^a^^,^^c^	−2.3 ± 7.1	11.9 ± 10.5^a^^,^^c^
MES/BW	Pre	0.21 ± 0.05	0.18 ± 0.02	0.22 ± 0.06	0.21 ± 0.04
	Post	0.22 ± 0.05	0.20 ± 0.03	0.34 ± 0.12*	0.24 ± 0.02
	Difference (%)	3.7 ± 15.3	10.9 ± 22.8	41.4 ± 31.3^a^^,^^b^^,^^d^	18.1 ± 30.5
MFS/MES Ratio	Pre	5.84 ± 2.01	7.02 ± 1.14	4.66 ± 1.04	5.95 ± 1.11
	Post	5.96 ± 2.33	6.93 ± 0.93	3.16 ± 1.39	5.72 ± 0.78
	Difference (%)	1.6 ± 15.9	0.9 ± 17.5	−27.8 ± 17.6	−1.9 ± 16.1

^a^ Statistical difference with CTRL (*p *< 0.05).

^b^ Statistical difference with TFlex (*p *< 0.05).

^c^ Statistical difference with Text (*p *< 0.05).

^d^ Statistical difference with TPaired (*p *< 0.05).

* Statistical difference between pre- and post-tests (*p *< 0.05).

## 4. Discussion

The aim of the study was to investigate antagonist muscle adaptation in climbers from the point of view of muscular capabilities. A first objective was to propose an easy-to-perform test to assess the flexor-to-extensor imbalance in climbers’ fingers and to observe the strength profiles in their fingers. The second objective was to explore the effectiveness of different types of training on performance and on rebalancing the flexor-to-extensor ratio.

### 4.1. Effect of level of expertise on capabilities and imbalance in fingers

Our results allowed us to determine a finger strength profile for the climbers, as well as ratio classes that allow us to measure the degree of imbalance between finger flexor and extensor strengths. Our results showed a positive correlation between the finger flexor capacity (MFS) with the climbing level which is in line with previous studies (8). As our sample was mixed-gender, the analysis enabled us to measure any gender-related effect. As no differences were observed between men and women when strength was normalised by body weight, the rest of the analysis was based on pooled data. The gender effect in our study differs from the literature, as Mermier et al. (31) found a higher strength in men than in women, despite body mass normalisation. In their study, the gender difference was explained by a lower climbing level in female participants compared with male participants. However, in our study, the climbing level of women (18.4 ± 4.1) was also lower (*p *= 0.013) than that of men (21.0 ± 4.1). Faced with this problem, we used ANCOVA with climbing level as a co-variate to correct for its effect on the variables analysed. This statistical approach may thus explain the different conclusion compared with Mermier et al. (31) who only performed a t-test without considering the effect of the climbing level. Future studies should thus take into account the climbing level as a co-variate to isolate the main effect of the factors tested and provide robustness in any conclusions.

Contrary to the results for MFS, MES results were not correlated with the climbing level which is in line with the literature (5, 8, 14). This confirms previous findings by Vigouroux et al. (14), who showed that practising climbing develops primarily the flexors, so it is justified to ask whether the balance of the finger flexor-to-extensor ratio should be shifted, especially given the complexity of the hand, which requires the intricate balancing of a whole chain of joints. This equilibrium implies a major action of the finger extensors, as previously shown in other types of grip (32, 33) which, without appropriate capacity, can limit finger force-generating capacity (34).

With regard to the MFS and MES results, the averaged MFS/MES ratio showed a strong imbalance in both men and women which is correlated with the climbing grade level. In our study the ratio revealed that the finger flexors were on average 6.27 times stronger than the finger extensors. This result is similar to the ratio previously observed in the literature for climbers [6.10 in Vigouroux et al. (14)]. A relationship between the MFS/MES ratio and climbing level was also shown, so the more experienced the climber, the more unbalanced the ratio, and the higher the need to rebalance the extensors' capacity. The histogram (Figure 2) provides ratio values that allow the imbalance to be considered and classified. For example, a climber with a ratio in the class of 6 (6.02–6.95) could be considered a “standard” climber (where the confidence interval is included). The “extreme” climbers (with a ratio higher than 8.75 defined by the *Z*-score) represented 7.7% of our participants, and are included in the two highest classes of MFS/MES ratio. With such imbalanced results, it is legitimate for climbers and trainers alike to decide whether a rebalancing should be undertaken since the extensors are highly solicited during climbing grips and such an imbalance could either limit performance or lead to overuse and injuries.

The main contribution of this first part is the easy-to-implement method which allows discriminating climbers from a muscular imbalance perspective. Although this method was based on external fingertip force measurements the results were in line with previous studies relying on more complex measurements and evaluating internal muscle capacities, confirming the validity of the present protocol. The main interest is that this method, unlike the one based on modelling by Vigouroux et al. (14), can be implemented in gyms for trainers and climbers. Given the complexity of the biomechanics (23 joint degrees of freedom) and muscles of the hand (more than 40 muscles), determining the capabilities of each muscle does indeed require a modelling approach using electromyography and kinematics, combining efforts on all the 23 joints of the hand under different force application conditions. This time-consuming method would not have been applicable to be consistent with our first objective and to use in daily training.

Few studies have focused on the flexor-to-extensor ratio in the upper limb of climbers, particularly in the shoulders and elbows (30, 31), and some differences have been found compared with non-climbers, but the impact of these consequences on the risk of injury needs to be confirmed as the climbers tested were all uninjured. Based on the method currently proposed, further studies are now needed to establish relationships between the occurrence of finger injuries and the value of the MES/MFS ratio, in order to investigate the pertinence of this ratio in the occurrence of injuries.

### 4.2. Effect of type of training on capabilities and imbalance in fingers

First of all, similar values in the control group between both pre- and post-tests showed that differences observed in other training groups are not attributed to a familiarization effect with the tests nor to other concomitant activities. Regarding the training effects, the hypothesis that flexor-based training increases MFS was confirmed by our results, which indicated an increase in MFS (+8.4% in the TFlex group and +11.9% in the TPaired group, on average). However, the hypothesis that extensor-based training increases MFS and MES was only partially confirmed: paired training increased MFS but not significantly MES (+18.1%), whereas extensor-only training increased MES (+41.4% in the TExt group on average) but not MFS. Thus, the MFS/MES ratio had a tendency to decrease with the extensor-only training (−27.8%) but seemed to remain stable in the other groups (between −1.9% and +1.6%). The increase in MFS after flexor-based training is in agreement with the literature (22, 26, 27, 35). The training with 80% MFS tested in the current study led to an 8.4% increase in strength. These improvements have been discussed in detail by Devise et al. (22) for this type of training. Briefly, the physiological phenomena activated are probably a combination of neural adaptation processes and metabolic stress that may be effective in increasing muscle strength.

No increase in MFS was observed in the extensor-only training. This differs from the literature focused on other joints, which showed an increase in hand grip (23) and elbow flexion (24) with antagonist training. This difference might be explained by several factors. First, the muscles analysed were not the same, especially as the fingers are at the end of the upper limb chain, so the adaptations may be different. In large muscles, hypertrophy can partly explain a strength gain, but the volume available in the forearms for the finger muscles is more limited and suggests more difficulties for development, which may explain the lack of increase (36). Secondly, the duration of our training protocols was shorter than in previous studies (4 vs. 6 weeks or more) and we can suppose that an increase may appear with a longer training program. Finally, climbers already have a higher initial flexor strength compared with non-climbers, which makes it more difficult to gain strength (22), whereas the population tested in the previous studies (23, 24) were untrained subjects. Thus, these effects would depend on the type of population studied, and it would appear that agonists in a trained population (i.e., with higher initial strength) would be less sensitive to strength gain.

The increase in MFS in the paired training is consistent with the literature concerning the APS training (28). This type of training was chosen because it might be beneficial for both strength development and injury prevention. As this type of training is an alternation of exercises involving the coupling of agonists and antagonists, it has the advantage of enhancing acute performance on agonists in a relatively short period of time [significant effects after 4-weeks of training (24)] and to be less-time consuming than traditional resistance training. Reported effects on antagonist strength are rarer but improvements may be expected as a previous study (37) has shown an increase in both flexor and extensor forearm strength in recreationally trained individuals. However, no significant increase in MES was observed in the paired training of our study, despite an average increase of 18.1%, which could be attributed to relatively high inter-individual variability. As MES is not correlated with climbing level, it cannot be the type of population (with a potentially higher initial MES) that affects our result. However, our results are similar to those of Fink et al. (38) who found no increase in one repetition-maximum for triceps, although the significance of their findings was questioned due to relatively large confidence intervals. It may also be that our training was not sufficiently optimum to be significant, but could probably be improved by simply changing the volume and/or rest periods during the training sessions.

There seemed to be a tendency for the MFS/MES ratio to decrease in the extensor-only training (−27.8%), due to a significant increase in MES without an increase in MFS. Again, the variability was relatively high. The mean ratio after the extensor-only training (3.16) was 47% lower than in the control group, close to or even lower than that found in non-climbers in the literature [3.66 in Vigouroux et al. (14)]. It can be assumed that extensor-based training may activate the same physiological phenomena as flexor-based training and as mentioned above. The effects of extensor-based training should be confirmed by an intervention longer than 4 weeks or with a higher training volume.

In the other training groups (the TFlex and TPaired groups), the MFS/MES ratio did not decrease so the flexor-to-extensor imbalance remained high. Although MFS increased, the ratio did not increase either, which means that MES must increase slightly, not enough to be significant but enough to keep the ratio similar. A certain amount of work was therefore done by the co-contraction of the finger extensors, which are the antagonist muscles, and are involved in the maintenance and stability of the joints (14, 33). However, the additional work on the extensors in the paired training was not sufficient as it did not increase the MES: it seems better to separate the training of the flexors from that of the extensors in order to obtain the best benefits.

From a practical point of view, the main conclusion is that improving MES is not obvious. Even if the extensors are highly engaged during climbing grip, TFlex or TPaired training is not suitable for improving their level. Only the TExt training over four weeks has been validated to rapidly enhance MES. Further studies with a longer training period should be conducted to explore whether this has a significant effect on the finger flexor-to-extensor ratio. In addition, the training volume of the TExt is only 15 min per session, so that it can be quickly and easily incorporated into a “classic” climbing training routine, making it potentially acceptable to climbers. It should be noted that the load applied in our study (>70% MES) is of high intensity to produce MES benefits. This training intensity is probably higher than that used in the popular exercise relying on elastic bands to train finger extensors. The amount of force exerted with elastic bands is not known and not constant throughout the extension phase. Unfortunately, to our knowledge, no studies have reported information on the effects of elastic band training on MES, but it can be expected that this exercise does not produce sufficient resistance and intensity to improve strength benefits (39).

### 4.3. Limitations and perspectives

This study presents some inherent limitations that should be considered. First of all, our results should be confirmed with higher-elite climbers as we only tested climbers from intermediate to elite climbers. In addition, our study lacked a population of non-climbers to exactly understand the adaptations associated with climbing. As a further analysis, it would be interesting to investigate the level of activation of the finger flexors and extensors before and after training using electromyography. This would highlight the neuromuscular adaptations that may have occurred and clarify the mechanisms that explain the strength gains whereas, in the current study, only assumptions of the phenomena can be made. In addition, the relationship between the MFS/MES ratio and the injury rate is only speculative given the current state-of-the-art. Further studies should thus focus on measuring the finger strength profile of previously injured climbers to provide more information. Furthermore, conducting a longitudinal study of climbers who have undergone rebalancing training and those who have not, and then observing the incidence of injury in both groups using the proposed assessment method would be a step forward in understanding injury prevention. Future research is therefore needed on this topic.

## 5. Conclusion

Our study proposed an easy-to-implement method and provided the basis for some reference values for finger strength, especially in the extensors. It has made it possible to classify climbers according to their MFS/MES ratio, which can help climbers and trainers to assess climbers and personalise training. The results obtained suggest that climbing at higher grade levels is associated with an increasingly imbalanced flexor-to-extensor ratio in climbers. Finally, our results showed that training the finger flexors increased the MFS and left the same imbalance as it does not benefit the extensor muscle groups. On the other hand, combining some flexor-extensor training in the way we did (combined in the same training exercise) only improved the MFS. On the contrary, extensor-only training improved extensor capacities and thus reduced the flexor-to-extensor imbalance, but this reduction did not lead to any improvement in maximum finger strength. Although further studies are required, the results of this study thus suggest that exclusively utilizing extensor-based training shows promise in reducing the flexor-to-extensor imbalance. This study was a first step in exploring the issue of antagonist muscle adaptation in climbers and therefore provided the basis for assessment and training to further investigate the potential implication of hand extensor strength and flexor-to-extensor imbalance on injury prevention and performance.

## Data Availability

The raw data supporting the conclusions of this article will be made available by the authors, without undue reservation.
